# Deep learning based searching approach for RDF graphs

**DOI:** 10.1371/journal.pone.0230500

**Published:** 2020-03-23

**Authors:** Hatem Soliman

**Affiliations:** College of Computer Science & Technology, Nanjing University of Aeronautics and Astronautics, Nanjing, China; Polytechnical Universidad de Madrid, SPAIN

## Abstract

The Internet is a remarkably complex technical system. Its rapid growth has also brought technical issues such as problems to information retrieval. Search engines retrieve requested information based on the provided keywords. Consequently, it is difficult to accurately find the required information without understanding the syntax and semantics of the content. Multiple approaches are proposed to resolve this problem by employing the semantic web and linked data techniques. Such approaches serialize the content using the Resource Description Framework (RDF) and execute the queries using SPARQL to resolve the problem. However, an exact match between RDF content and query structure is required. Although, it improves the keyword-based search; however, it does not provide probabilistic reasoning to find the semantic relationship between the queries and their results. From this perspective, in this paper, we propose a deep learning-based approach for searching RDF graphs. The proposed approach treats document requests as a classification problem. First, we preprocess the RDF graphs to convert them into N-Triples format. Second, *bag-of-words* (BOW) and *word2vec* feature modeling techniques are combined for a novel deep representation of RDF graphs. The attention mechanism enables the proposed approach to understand the semantic between RDF graphs. Third, we train a convolutional neural network for the accurate retrieval of RDF graphs using the deep representation. We employ 10-fold cross-validation to evaluate the proposed approach. The results show that the proposed approach is accurate and surpasses the state-of-the-art. The average *accuracy*, *precision*, *recall*, and *f-measure* are up to *97.12%*, *98.17%*, *95.56%*, and *96.85%*, respectively.

## 1 Introduction

The digital age arrives with a set of challenges for the Web due to the abundance of information. In today’s modern society, people capture, upload, store, and digitalize almost every activity of daily life routine over the Web. Nowadays, communication devices have the capacity to connect to the internet independently and contain sensors that are spreading useful information without users’ intervention. Consequently, the data is increasing daily and resulting in *information overload*. Searching such data had driven to the development of the linked data and Semantic Web. It considers the *machine processable* metadata [1] that enhances information flow and can relate data from distributed data sources to make data meaningful. This mash-up of data introduced the phrase *Web 3.0*. Building links between distributed data sources is essential to Web 3.0 which is achieved using RDF. RDF resources consist of RDF triples where each triple contains a subject *s* that has property *p* with value *o* [2, 3]. Consequently, a RDF triple may be viewed as a representation of an atomic *fact* or a *claim* [4].

The analogous datasets may be defined as *Linked Data* [5] that can be summarized as being “*simply about using the Web to create typed links between data from different sources*”. Linked data combine entities from different sources/locations to crawl them as a data-space due to its connected links [5, 6]. This idea motivates this study to access the required information from distributed sources and build links that help in searching. RDF triples allow entities to be queried and linked together. The existing studies use RDF and SPARQL to serialize the content and to execute the queries for searching, respectively.

RDFs are massive in size and crucial; therefore it is not easy to extract information for an ordinary user. Although, linked data and SPARQL provides a significant improvement in search methods. However, the complexity criteria (similar triples by RSFS and OWL rules) and the usability criteria (the human effort) are required to read and learn RDF data [7]. For example, SPARQL queries require structure accuracy to extract RDF elements. Such queries do not allow the statistic analysis to evaluate the query against the RDF content; e.g., features of a basket may not be enough as input to identify the *online shopping basket*. Many approaches have been proposed for achieving this kind of searching from RDF data using linked data and SPARQL [8–19]. Notably, such approaches respond to queries with an exact match rather than estimating the similarity within the RDF content that leads to the original motivation for the work in this paper. On the other hand, Hadi *et al*. [20] exploited a machine learning approach to search RDF graphs. Although their approach works on statistical estimation, it does not consider semantic relationships while searching RDF graphs and requires significant improvement.

To this end, a deep learning-based searching approach is proposed for RDF graphs. In this regard, we first reuse the history-data of *DBpedia* documents. Second, we preprocess the extracted RDF graphs using the W3C validation service. Third, we concatenate *BOW* and *word2vec* feature modeling techniques for attention-based recurrent neural network novel deep representation of RDF graphs. The attention mechanism enables the proposed approach to understand the semantic between RDF graphs. Fourth, we train a convolutional neural network for the accurate retrieval of RDF graphs using the deep representation. Finally, a convolutional neural network is trained to predict the retrieval of RDF graphs. And the proposed approach is evaluated using a 10-fold cross-validation technique on the given dataset. The evaluation results show the accuracy of the proposed approach. The average *accuracy*, *precision*, *recall*, and *f-measure* are up to *97.12%*, *98.17%*, *95.56%*, and *96.85%*, respectively.

The main contributions of this study are as follows:

- An approach based on a convolutional neural network is proposed for searching RDF graphs. To the best of our knowledge, we are the first to exploit a deep learning algorithm in retrieval prediction of RDF graphs.
- Evaluation results of the proposed approach on the given dataset show that the proposed convolutional neural network-based approach is accurate and surpasses the state-of-the-art.

The rest of the paper is organized as follows: Section II presents the proposed approach. The evaluation process and results of the proposed approach are described in Section III. Section IV explains the threats. Section V and Section VI present the related work and conclusion, respectively.

## 2 Approach

### 2.1 Overview

**Fig 1:**
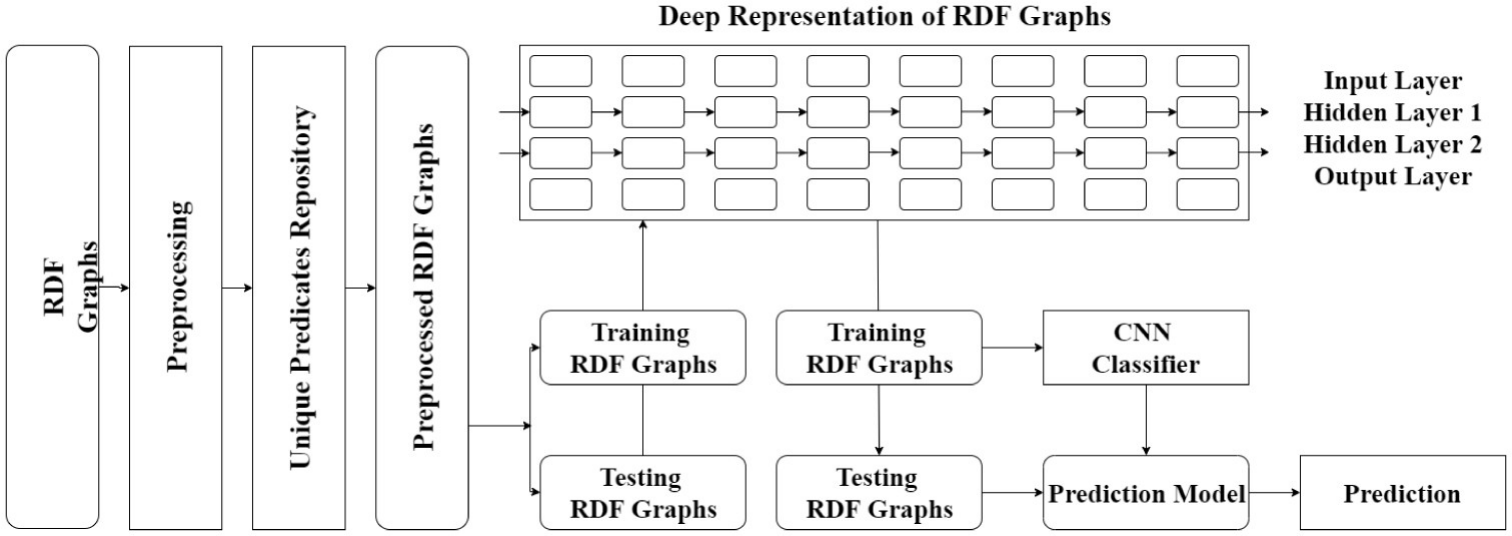
Overview of the proposed approach.

Fig 1 illustrates an overview of the convolutional neural network based searching for RDF graphs. The proposed approach performs RDF graph retrieval prediction as follows:

1. We reuse the history-data of RDF graphs as training data.
2. We apply the W3C validation service to RDF graphs for preprocessing.
3. We concatenate *BOW* and *word2vec* feature modeling techniques for a novel deep representation of RDF graphs.
4. A convolutional neural network is trained to anticipate the retrieval of RDF graphs. We pass the deep representation to the classifier as input that predicts the retrieval of RDF graphs.

The following sections introduce the key steps of the proposed approach.

### 2.2 Illustrating example

**Fig 2:**
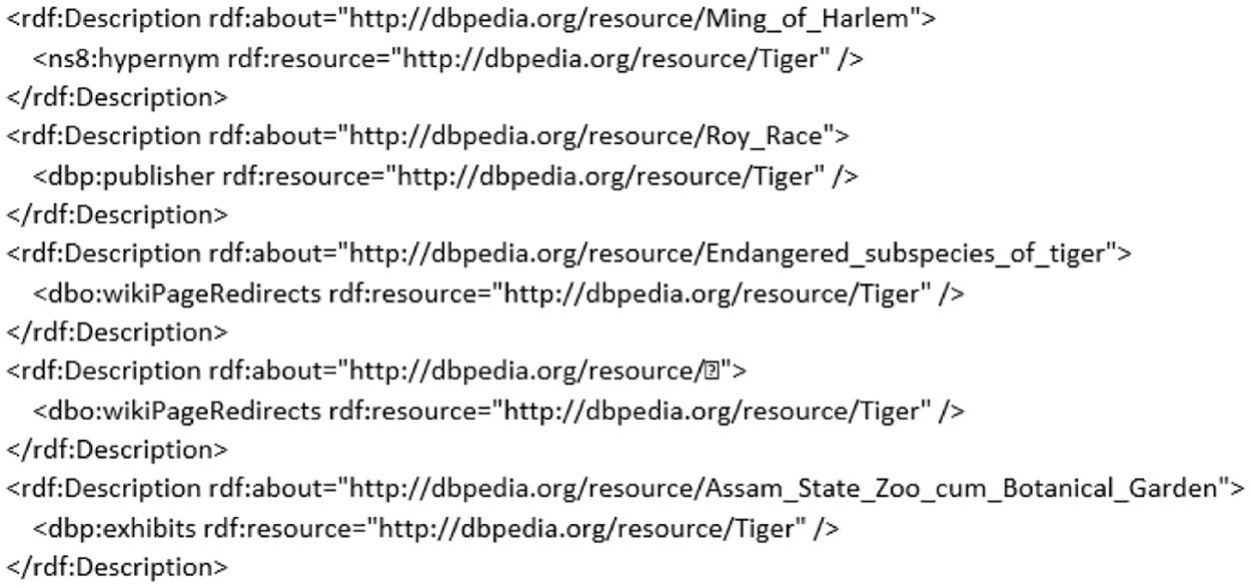
An example of RDF graph.

We consider an example to demonstrate how the proposed approach anticipates the retrieval of RDF graphs. An excerpt of RDF graph taken from *DBpedia* is presented in Fig 2. The details on how the proposed approach deals the illustrating example are given in the following sections.

### 2.3 Problem definition

A RDF *d* is a graph from a set of RDF graphs (D) which can be formalized as,

$$\begin{array}{c} d=\;<t_{1},t_{2},\ldots ,t_{n}> \end{array}$$ (1)

where, *t*_1_, *t*_2_, …, *t*_*n*_ represents the *n* number of triples in a RDF graph, and each triple consists of *subject*(*s*), *predicate*(*p*), and *object*(*o*).

$$\begin{array}{c} d_{e}=\;<t_{e_{1}},t_{e_{2}},\ldots ,t_{e_{n}}> \end{array}$$ (2)

where,

*d*_*e*_ = a complete example of a RDF graph represented in Fig 2,

$t_{e_{1}}$ = first triple of the example,

$t_{e_{2}}$ = second triple of the example,

.

.

$t_{e_{n}}$ = last triple of the example.

The proposed approach takes the problem of searching of RDF graphs as classification problem and predicts whether a RDF graph will be retrieved or not. The retrieval anticipation of a new RDF graph *d* can be defined as a function *f*.

$$\begin{array}{c} c=f\left(d\right)\;c\in \{hit\;or\;miss\},\;d\in D \end{array}$$ (3)

where, *c*, *f*, *d*, and *D* are predefined classification (*hit* or *miss*), classification function of retrieval anticipation, a RDF graph, and a set of RDF graphs, respectively.

### 2.4 Preprocessing

We preprocess each of the RDF graph using the *W3C Validation Service*. We load each collected RDF graph using *Apache Jena API* (http://jena.apache.org/) to validate its syntax. Then, the validated RDF graph is loaded into the model that transforms the RDFs from *serialization* format to *N-Triples* format. The preprocessing of a RDF graph can be formalized as,

$$\begin{array}{c} d=\;<{t_{1}}^{{}^{\prime }},{t_{2}}^{{}^{\prime }},\ldots ,{t_{n}}^{{}^{\prime }}> \end{array}$$ (4)

where, ${t_{1}}^{{}^{\prime }},{t_{2}}^{{}^{\prime }},\ldots ,{t_{n}}^{{}^{\prime }}$ are preprocessed *n* triples of the RDF graph *d*.

**Fig 3:**
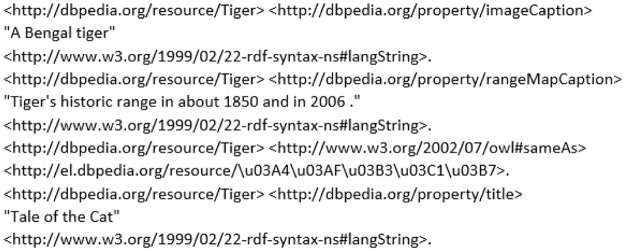
Preprocessed example of RDF graph.

For the motivating example presented in Section 2.2, a RDF graph *d*_*e*_ after preprocessing can be formalized as,

$$\begin{array}{c} d_{e}=\;<{t_{e_{1}}}^{{}^{\prime }},{t_{e_{2}}}^{{}^{\prime }},\ldots ,{t_{e_{n}}}^{{}^{\prime }}> \end{array}$$ (5)

where, from the excerpt of preprocessed RDF graph is presented in Fig 3, ${t_{e_{1}}}^{{}^{\prime }},{t_{e_{2}}}^{{}^{\prime }},\ldots ,{t_{e_{n}}}^{{}^{\prime }}$ represent the *n* triples (separated with a dot (.)) of the preprocessed RDF graph, respectively.

### 2.5 Deep feature representation

**Fig 4:**
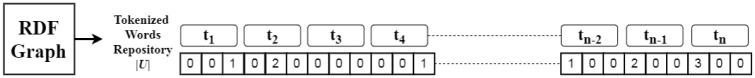
Traditional feature representation model.

**Fig 5:**
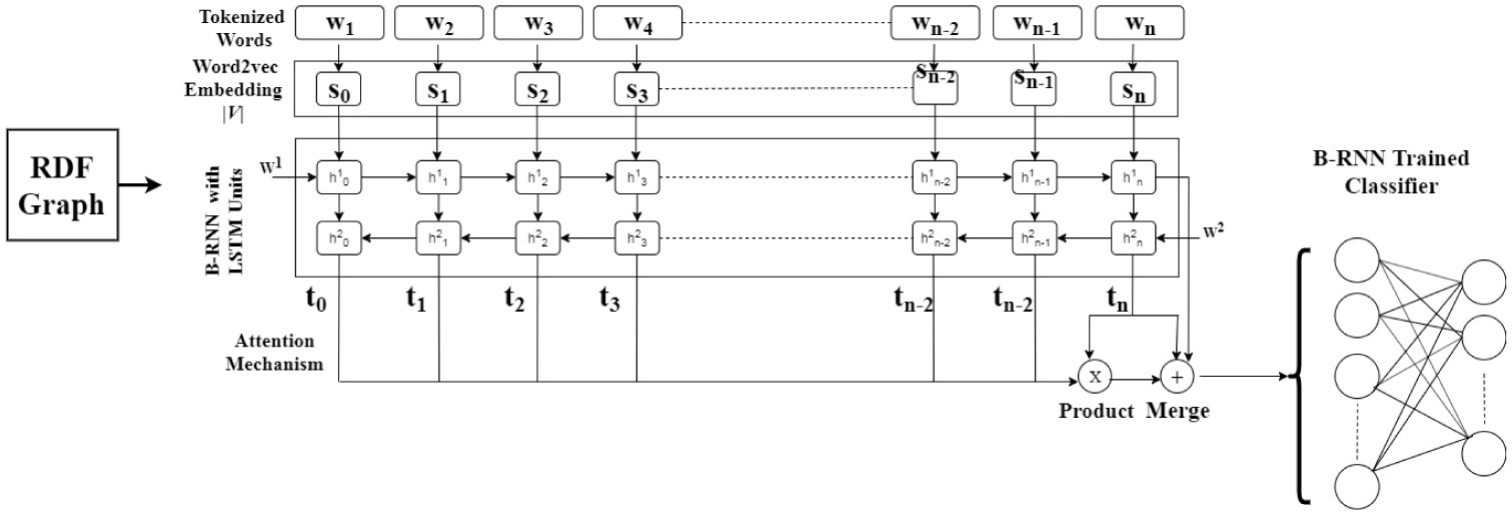
Deep representation model.

A *BOW* representation of each RDF graph provides a boolean (0 or 1) array or a term frequency array using all repository terms [21] (shown in Fig 4) and does not incorporate the semantic similarity among terms. Moreover, problems like high dimensionality and sparse data are observed in the bag-of-n-words feature representation [22]. To this end, a neural network-based features representation model (*word2vec*) is proposed to learn and understand the semantic relationship between terms (predicates in our case) [23]. However, *word2vec* only considers semantics of individual terms rather than a sequence of words. Notably, a significant improvement is required to combine the sequence of terms, the syntax of terms, the semantic relationship among terms. In this perspective, a deep representation of RDF graphs is proposed. Fig 5 illustrates an overview of the deep representation of RDF graphs. The long short term memory (LSTM) cells are exploited [24] as a memory unit in the hidden layer that resolve the vanishing gradient problem [25]. LSTM cells can memorize the sequence of terms in both forward direction and backward direction.

The construction of deep representation involves the extraction of |*U*|- dimensional representation (*BOW*) using predicate repository, the learning of |*V*|-dimensional representation *word2vec* using |*U*|-dimensional representation, and the learning of LSTM cells (deep representation) using |*S*|-dimensional representation. This process returns the |*D*|-dimensional representation of the given RDF graph. The |*D*|-dimensional representation has a sequence network (recurrent neural network) that contains a hidden layer with *n* hidden units (*h* = *h*_1_, *h*_2_, …‥, *h*_*n*_). The recurrent neural network takes |*V*|-dimensional representation (*y* = *y*_1_, *y*_2_, …‥, *y*_*n*_) as an input and returns a |*D*|-dimensional representation (*z* = *z*_1_, *z*_2_, …‥, *z*_*n*_). Every *h* transforms the previous state *s*_*i*−1_ and a term *y*_*i*_ into the next state *s*_*i*_ and output word *z*_*i*_. Every hidden unit repeatedly performs the function *f* in recurrent neural network:

$$\begin{array}{c} f:\;\{s_{i-1},y_{i}\}\to \;\{s_{i},z_{i}\} \end{array}$$ (6)

where each *s*_*i*_ has the information of *i*^*th*^ term in *h*, whereas the output *z*_*n*_ of *h*_*n*_ represents the complete RDF graph.

Additionally, an attention mechanism is employed to learn from the predicates of the RDF graph. An attention vector with the weighted summation of all outputs *z*_*i*_ can be formalized as,

$$\begin{array}{c} a_{n}=\sum_{i=1}^{n}\alpha_{i}z_{i} \end{array}$$ (7)

where *α*_*i*_ is the weight of each word *y*_*i*_ that defines the importance of *y*_*i*_ for classification. A bidirectional recurrent neural network learns representation with input word sequence (forward and backward). A complete deep representation of *d* can be formalized as,

$$\begin{array}{c} d=z_{n}+a_{n}+a_{n}+z_{n} \end{array}$$ (8)

where, + represents the concatenation of vectors.

The hyper-settings of the proposed deep representation model as follows: 300 LSTM units, 0.2 dropout probability, 0.001 learning rate, and *binary* *cross*-*entropy* based loss function with *Adam* *optimizer*. We set 100 epochs for the training. Notably, the proposed model is implemented in *Python Keras Library* [26]. To the best of our knowledge, we are the first to apply deep representation to learn the RDF graph representation. We use deep representation to train a convolutional neural network for the retrieval anticipation of RDF graphs.

### 2.6 Deep learning classifier

**Fig 6:**
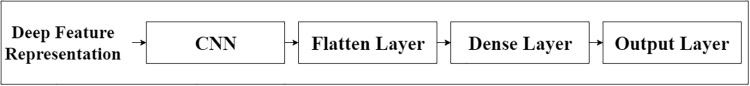
Overview of the deep learning-based classifier.

Fig 6 illustrates an overview of deep learning classifier. We leverage the convolutional neural network for retrieval prediction of RDF graphs. We select the convolutional neural network because of the following reasons: 1) its deep semantic relationship learning capabilities among words [27]; 2) it avoids the gradient problem of recurrent neural network [28] by applying different filter sizes.

To train the convolutional neural network, the deep representation is forwarded to convolutional neural network that contains 3 layers of CNN, filter 128, kernel size 1, loss function *binary*-*crossentropy*, and activation *tanh*. Then, the output of the convolutional neural network is passed to a flatten layer [29] that returns a 1-dimensional vector. Finally, the dense layer connects the neurons between layers and the output layer returns the retrieval prediction of RDF graphs.

## 3 Evaluation

This section defines the research questions to evaluate the proposed approach, explains how RDF graphs are collected, presents the metrics and evaluation process of the proposed approach, and discusses the results while answering the research questions.

### 3.1 Research questions

The proposed approach is evaluated by investigating the following research questions:

- RQ1: How accurate the proposed approach in retrieval prediction of RDF graphs?
- RQ2: Does the proposed classifier outperform other machine/deep learning classifiers in retrieval prediction of RDF graphs?
- RQ3: Does features’ preprocessing influence in predicting the retrieval of RDF graphs?

The *RQ1* examines the accuracy of the proposed approach. In this perspective, the proposed approach is compared with the state-of-the-art approaches: a graph-based retrieval of RDFs (GRSearch) [30] and machine learning-based retrieval of RDFs (MLSearch) [20]. We also compare the proposed approach with the two baseline algorithms: *random prediction algorithm* and *zero-rule algorithm* to double-check the performance of the proposed approach.

The *RQ2* compares the performances of different machine/deep learning classifiers to reveal whether the proposed approach outperforms other machine/deep learning classifiers in retrieval prediction of RDF graphs.

The *RQ3* examines the influence of the features’ preprocessing. In this perspective, we compute and compare the performance of the proposed approach with and without preprocessing.

### 3.2 Dataset

We collect the *DBpedia* dataset (https://wiki.dbpedia.org/data-set-30). DBpedia 2016-10 release contains 13 billion pieces of information out of which 1.7 billion were extracted from the English edition of Wikipedia. We use only 1.7 billion RDF triples (English version) to evaluate the proposed approach; however, we ignore all syntactically invalid triples, as mentioned in Section 2.4. Note that we divide the data into four different search categories: *Triple-pattern requests with multiple responses; e.g., British actors and their birth regions*, *Extended triple-pattern requests with multiple responses; e.g., Movies having award-winning feminist actors*, *Triple-pattern requests with zero responses; e.g., MIT graduates born in Steve Jobs’s death place*, and *Extended triple pattern requests with zero responses; e.g., People who influenced by Egyptian writers* to evaluate the proposed approach.

### 3.3 Process and metrics

Algorithm 1 shows the process to compute the best classifier (*CNN*) as mentioned in Section 2. Algorithm 1 consists of three parts. In the first part (Line 1), we set cross-validation (sometimes called rotation estimation) [31] *M* on *D*. We divide *D* into ten segments notated as *M*_*i*_(*i* = 1, 2, …., 10). We subtract the RDF graphs that belong to *M*_*i*_ and mark them as testing RDF graphs *Test*, and the remaining RDF graphs are marked as training RDF graph *Train*. In the second phase (Lines 2-11), we apply the *M*-fold cross-validation and train/test the classifiers (MNB, LR, RF, SVM, LSTM, and CNN). For each iteration of cross-validation, we first separate the training dataset *Train* and testing dataset *Test* (Line 3). Then, we train the classifiers with *Train* and evaluate each classifier with *Test* (Lines 4-10). In the last phase (Lines 12-13), we compute and compare the metrics (*accuracy*, *precision*, *recall*, and *F1*) of each classifier, and return the best classifier.

**Algorithm 1 Identification of Best Machine/Deep Learning Algorithm for the Proposed Approach**

Input: DBpedia RDF graphs *D*

Output: Best machine learning classifier for Searching ⊆ *D* from *D*

1: *M*_*i*_(*i* = 1, 2, 3, ……, 10); *Train* = *M*_*j*_; *Test* = *M*_*i*_; *j* ≠ *i*

2: **for** each cross-validation *m* from *M* **do**

3:  *Train*_*i*_ = ⋃_*i*∈[1,10]∧*j*≠*i*_ *M*_*j*_

4:  Train a Multinomial Naive Bayes (MNB) classifier with training data *Train*.

5:  Train a Logistic Regression (LR) classifier with training data *Train*.

6:  Train a Random Forest (RF) classifier with training data *Train*.

7:  Train a Support Vector Machine (SVM) classifier with training data *Train*.

8:  Train a Long Short Term Memory (LSTM) classifier with training data *Train*.

9:  Train a Convolutional Neural Network (CNN) classifier with training data *Train*.

10:  Take the trained MNB, LR, RF, SVM, LSTM, and CNN for retrieval prediction of each RDF graph with testing data *Test*.

11: **end for**

12: Calculate the *accuracy*, *precision*, *recall*, and *F1* of MNB, LR, RF, SVM, LSTM, and CNN.

13: Compare their predicted value with actual value.

14: **return** Best Classifier.

The selected metrics are commonly adopted metrics for the performance evaluation of classification algorithms [32–37]. Therefore, we calculate the retrieval related *accuracy*, *precision*, *recall*, and *f-measure* for the performance evaluation of the proposed approach on the given RDF graphs that can be defined as,

$$\begin{array}{c} Acc=\frac{TP\;+\;TN}{TP\;+\;TN\;+\;FP\;+\;FN} \end{array}$$ (9)

$$\begin{array}{c} Pre=\frac{TP}{TP\;+\;FP} \end{array}$$ (10)

$$\begin{array}{c} Rec=\frac{TP}{TP\;+\;FN} \end{array}$$ (11)

$$\begin{array}{c} F1=\frac{2\;*\;Pre\;*\;Rec}{Pre\;+\;Rec} \end{array}$$ (12)

where, *Acc*, *Pre*, *Rec*, and *F1* represent the *accuracy*, *precision*, *recall*, and *f-measure* of the proposed approach in retrieval prediction of RDF graphs, respectively. *TP* represents the number of RDF graphs that the proposed approach predicts correctly as *hit*, *TN* represents the number of RDF graphs that the proposed approach predicts correctly as *miss*, *FP* represents the number of RDF graphs that the proposed approach predicts incorrectly as *hit*, and *FN* represents the number of RDF graphs that the proposed approach predicts incorrectly as *miss*.

### 3.4 Results

#### RQ1: Accuracy of the proposed approach

We answer the **RQ1** by performing a comparison between the proposed approach and the state-of-the-art approaches: MLSearch and GRSearch. We also compare the proposed approach with a random prediction algorithm and a zero-rule algorithm. We consider both algorithms because the proposed approach is the first approach to leverage deep learning algorithms for retrieval prediction of RDF graphs.

The evaluation results of the proposed approach and the baseline approaches are presented in Table 1. Approaches are presented in the first column of the table. The results of performance metrics (*Acc*, *Pre*, *Rec*, and *F1*) for each classifier are presented in Columns 2-5 of the table, respectively. Each row of the table presents the performance of the corresponding approach, respectively. The average *Acc*, *Pre*, *Rec*, and *F1* of the proposed approach, MLSearch, and GRSearch are (*97.12%*, *85.21%*, and *78.63%*), (*98.17%*, *87.52%*, and *69.20%*), (*95.56%*, *79.19%*, and *66.31%*), and (*96.85%*, *83.15%*, and *67.72*), respectively.

**Table 1:** Comparison against baseline approaches.

	Acc	Pre	Rec	F1
Proposed Approach	97.12%	98.17%	95.56%	96.85%
MLSearch	85.21%	87.52%	79.19%	83.15%
GRSearch	78.63%	69.20%	66.31%	67.72%

Table 2 shows the evaluation results of random prediction, zero-rule, and the proposed approach. Approaches are presented in the first column of the table. The results of performance metrics (*Acc*, *Pre*, *Rec*, and *F1*) for each classifier are presented in Columns 2-5 of the table, respectively. The rows of the table present the performance of the approaches, respectively. The average *Acc*, *Pre*, *Rec*, and *F1* of the proposed approach, random prediction, and zero-rule are (*97.12%*, *65.40%*, and *87.62%*), (*98.17%*, *65.93%*, and *80.12%*), (*95.56%*, *55.56%*, and *83.26%*), and (*96.85%*, *60.36%*, and *81.66*), respectively.

**Table 2:** Comparison against random prediction and zero-rule.

	Acc	Pre	Rec	F1
Proposed Approach	97.12%	98.17%	95.56%	96.85%
Random Prediction	65.40%	65.93%	55.65%	60.36%
Zero-rule	87.62%	80.12%	83.26%	81.66%

The observations from Tables 1 and 2 are as follows:

- The proposed approach outperforms the baseline approaches, random prediction, and zero-rule classifiers in *Acc*, *Pre*, *Rec*, and *F1*, respectively.
- The improvement of the proposed approach upon MLSearch in *Acc* and *F1* is *13.98% = (97.12%—85.21%) / 85.21%* and *16.52% = (96.85%—83.12%) / 83.12%*, respectively.
- The improvement of the proposed approach upon GRSearch in *Acc* and *F1* is *23.52% = (97.12%—78.63%) / 78.63%* and *43.02% = (96.85%—67.72%) / 67.72%*, respectively.
- The improvement in the performance of the proposed approach upon random prediction in *Acc* and *F1* is *40.86% = (92.12%—65.40%) / 65.40%* and *57.17% = (94.86%—60.36%) / 60.36%*, respectively.
- The improvement in the performance of the proposed approach upon zero-rule in *Acc* and *F1* is *5.13% = (92.12%—87.62%) / 87.62%* and *16.17% = (94.86%—81.66%) / 81.66%*, respectively.

**Fig 7:**
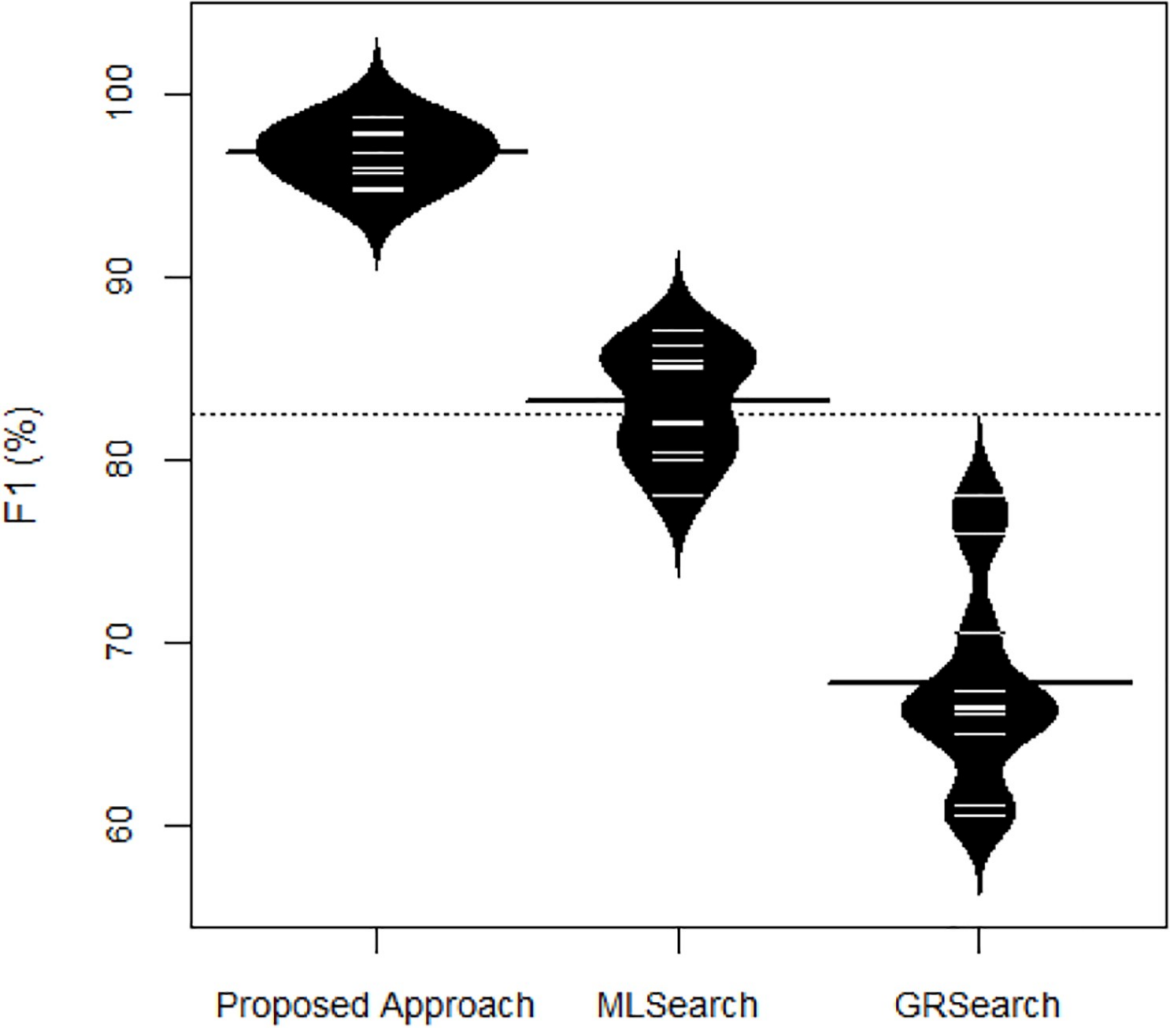
Distribution on accuracy.

We present the accuracy distribution of 10-fold cross-validation for the proposed approach and baseline approaches in Fig 7. We compare the *F1* distributions of each approach and plot one bean against each approach. Each short horizontal line within a bean illustrates the *F1* on a *i*^*th*^ fold, whereas the long horizontal line illustrates the average *F1*. We observe that the proposed approach outperforms the baseline approach in each fold. Notably, the average *F1* of the proposed approach is significantly large as compared to the best performances of the baseline approach.

We also employ ANOVA (one-way) to confirm the significance of the proposed approach and the basline approach. It may examines whether the single factor (i.e., different approaches) is the only difference that drives to the difference in performance. Note that ANOVA is conducted independently on the *Acc*, *Pre*, *Rec*, and *F1*. Table 3 presents the results of ANOVA that shows *F* > *F*_*cric*_ and *P*_*Value*_ < *(alpha = 0.05)* are true for each *Acc*, *Pre*, *Rec*, and *F1*. It indicates that using different approach (the single factor) has a significant difference in the performances of both approaches. The preceding analysis concludes that the proposed approach is accurate in retrieval anticipation of RDF graphs.

**Table 3:** Results of ANOVA analysis.

Source of Variation	SS	df	MS	F	p-value	F-crit
***Acc***						
Between Approaches	0.02777	1.00000	0.02777	26.54565	0.00007	4.41387
Within Approaches	0.01883	18	0.00105			
Total	0.04659	19				
***Pre***						
Between Approaches	0.01746	1	0.01746	30.57721	0.00003	4.41387
Within Approaches	0.01028	18	0.00057			
Total	0.02774	19				
***Rec***						
Between Approaches	0.02696	1	0.02696	17.56680	0.00055	4.41387
Within Approaches	0.02762	18	0.00153			
Total	0.05458	19				
***F1***						
Between Approaches	0.02212	1	0.02212	27.75361	0.00005	4.41387
Within Approaches	0.01435	18	0.00080			
Total	0.03647	19				

#### RQ2: Performance comparison of machine/deep learning algorithms

We answer the **RQ2** by applying the most adopted machine/deep learning text classification algorithms (*CNN*, *LSTM*, *MNB*, *LR*, *RF*, and *SVM*) due to their competitive performance [27, 37–39]. The evaluation of the proposed approach with *SVM* yields most accurate results and outperforms the other classifiers on the given dataset.

Table 4 presents the evaluation results of *CNN*, *LSTM*, *SVM*, *LR*, *RF*, and *MNB*. The first column of the table presents the cross-validations. Columns 2-5 of the table present the performance results of *Acc*, *Pre*, *Rec*, and *F1* for each classifier, respectively. Rows of the table present the performance of a particular classifier, respectively.

**Table 4:** Comparison against machine learning algorithms.

ML Classifier	Acc	Pre	Rec	F1
CNN	97.12%	98.17%	95.56%	96.85%
LSTM	89.96%	91.06%	88.94%	89.99%
SVM	85.21%	87.52%	79.19%	83.15%
MNB	83.24%	77.14%	83.24%	80.07%
LR	90.54%	93.69%	95.15%	94.41%
RF	91.33%	92.27%	94.88%	93.56%

The average *Acc*, *Pre*, *Rec*, and *F1* of *CNN*, *LSTM*, *SNM*, *MNB*, *LR*, and *RF* are (*97.12%*, *89.96%*, *85.21%*, *83.24%*, *90.54%*, and *91.33%*), (*98.17%*, *91.06%*, *87.52%*, *77.14%*, *93.69%*, and *92.27%*), (*95.56%*, *88.94%*, *79.19%*, *83.24%*, *95.15%*, and *94.88%*), and (*96.85%*, *89.99%*, *83.15%*, *80.07%*, *94.41%*, and *93.56*), respectively.

The observations from the Table 4 are as follows:

- The *CNN* classifier surpasses all other classifiers in *accuracy*, *precision*, *recall* and *F1*. The reason is that *CNN* is better for extracting position invariant features as compared to *LSTM* and performs well with high dimensional feature sets [40].
- The *CNN* classifier surpasses all the other machine learning classifiers. It converts non-linearly classifiable and inter-dependent feature data into a higher-dimensional hyperplane if the classification of the data is not possible linearly.
- Although, the existing research [41] reports *MNB* classifier is effective in classification; however, it does not work well with the proposed approach on the given dataset. One possible reason is that the input predicates (features) to the classifier for training are inter-related, and *MNB* classifier performs well if the features are independent [27, 42]. The evaluation results of *MNB* on the given dataset are not effective as compared to *SVM*, *LR*, and *RF* with the proposed approach.
- The performance results of *LR* and *RF* are very close to the *SVM*. A larger dataset may reveal that one of them is better than *SVM*.

The preceding analysis concludes that *CNN* works better than the other classifiers with the proposed approach.

#### RQ3: Influence of features’ preprocessing

The different RDF graphs may have similar predicates (features) or may have superlative/comparative words in predicates. Passing such data as features to a machine learning algorithm is an overhead. It reduces performance and increases the computational cost of machine learning algorithms.

We answer the **RQ3** by performing the comparison between the evaluation results of the proposed approach with and without features’ preprocessing. The evaluation results are presented in Table 5. The preprocessing input settings are presented in the first column of the table. Columns 2-5 of the table present the performance results of *Acc*, *Pre*, *Rec*, and *F1*. The rows of the table present the performance of the proposed approach to the different settings of preprocessing, respectively. The improvement in the performance of the proposed approach with different preprocessing settings is presented in the last row of the table.

**Table 5:** Influence of preprocessing.

Preprocessing	Acc	Pre	Rec	F1
Enable	92.12%	94.17%	95.56%	94.86%
Disable	78.93%	82.33%	85.92%	84.08%
Improvement	**16.71%**	**14.38%**	**11.22%**	**12.82%**

From the Table 5, we make the following observations:

- The preprocessing enabled proposed approach achieves significant improvement in performance. The evaluation results suggest that the performance improvement in *Acc*, *Pre*, *Rec*, and *F1* are up to *16.71%*, *14.38%*, *11.22%*, and *12.82%*, respectively.
- The preprocessing disabled approach significantly decreases the *Rec* from *95.56%* to *85.92%*. The decrease in *Rec* returns incorrect results against the requested query. One possible reason of the decrease in performance is the similar or superlative/comparitive words in the predicates of the given triples.

The preceding analysis concludes that preprocessing of the features is essential to the proposed approach.

### 3.5 Threats to validity

There could be some elements that may affect the performance of the proposed approach. The followings are the threats to the validity of the proposed approach.

- The selection of evaluation metrics is the first threat to construct validity. We select *Acc*, *Pre*, *Rec*, and *F1* metrics for the evaluation of the proposed approach. Because, they are the most adopted metrics [32–37] for the evaluation of classification problems.
- The leverage of *NLTK* for the preprocessing of the extracted features (as mentioned in Section 2.5) is a threat to construct validity. We select *NLTK* due to its performance and popularity [37]. The use of any other natural language processing repository may affect the said results of the proposed approach.
- The generalizability of the proposed approach is a threat to external validity. We focus the RDF graphs from an open-source dataset (*DBpedia*) for the evaluation of the proposed approach. We cannot guarantee the results of the proposed approach with other datasets.

## 4 Related work

The WWW is an information space where RDF graphs and other web resources are identified by URLs that may be interlinked and are accessible over the Internet. It is difficult to get the right URLs against asked queries due to the information overload caused by the current digital era. To address this problem, Tim Burner Lee introduced the semantic web that provides a common framework and allows data to be shared and reused across applications. It considers semantics for searching rather than keyword matching and query responses. Linking data together from different resources is the key to the semantic web. Moreover, linking data is essential to connect and search data over the semantic web. Linked data rely on RDF graphs that contain data in RDF format. Many approaches have been proposed on the efficient search of RDF graphs. Such approaches mainly focus on classical RDF searching e.g., keyword-based searching or graph-based searching.

Tran et al. [43] introduced the idea of generating summary-graphs for the original RDF graph to generate and rank candidate SPARQL queries. Then, Zhang et al. [44] proposed a solution to this idea. Moreover, Yang et al. [45] proposed tree patterns to connect keywords specified by the users where the tree patterns are ordered by their size relevance, and Zheng et al. [46] proposed a method to search semantically equivalent structure patterns. Finally, De Virgilio [47] proposed an RDF keyword-based query vis Tensor calculus and later extended it to a distributed environment via MapReduce [48].

Nagarajan *et al*. [49] presented ontology-based multi-model semantic information retrieval system. It is based on the idea of integrating domain knowledge and images and retrieves the required multi-modal information using a fuzzy rule set. It also provides the image semantic by constructing visual words using the probabilistic latent semantic. Other researches [50–52] also proposed formalize and semantic visualization models based on the fuzzy rule set.

Nhuan *et al*. [53] proposed an approach that determines the degrees of equality between relations (properties) defined by different vocabularies. They consider the occurrences of matching pairs of RDF triples to find the intervals representing lower and upper levels of property equality. Consequently, they obtained a graph of similar properties where the interval-based strength of edges represents degrees of similarity between properties.

Jaafar *et al*. [54] proposed a fuzzy knowledge-based framework to realize a nature and visualized F-RDF retrieval operation, to help an end-user to enhance the querying and accessing Web data.

Gupta *et al*. [18] introduced a ranking function based on fuzzy logic to enhance Information Retrieval. The function based on the computation of term-weighting schemas such as term frequency, inverse document frequency, and normalization. The state-of-the-art [15–18] has described the difficulties in the understanding of a semantic search engine. The motive behind is to propose an approach based on RDF, and the automatic identification of content over the WWW.

As a conclusion, researchers have proposed different approaches [8–19, 55, 56] for retrieving information using RDF; however, it requires significant improvement. Moreover, none of them employs machine learning classification algorithms to address this problem. Notably, the proposed approach differs in that the existing approaches as we are first to apply the support vector machine for the retrieval of RDF graphs.

## 5 Conclusion

In this digital era, Web users share almost every moment of daily life on the Internet that causes information overload. Consequently, it is difficult to accurately retrieve the required information without understanding the syntax and semantics of the content. To this end, in this paper, a deep learning-based approach for searching RDF graphs is proposed that treats RDF graph requests as a classification problem. The proposed approach applies a deep learning classifier on the given dataset for the retrieval anticipation of RDF graphs. The proposed approach introduces a new way to search the RDF graphs and helps the Web users in answering their queries. We perform the 10-fold cross-validation for the evaluation of the proposed approach using the open-source RDF graphs of *DBpedia*. The evaluation results show that the proposed approach is accurate.

The broader impact of this study is to indicate that the triples in the RDF graphs are a rich source of information for accurate retrieval prediction of RDF graphs. Our results motivate future research on the retrieval anticipation of RDF graphs. We want to investigate a retrieval prediction of RDF graphs with a deep learning approach with deep hyperparameter settings. This will also confirm the generalizability of the proposed approach.
